# Structural and Dynamical Effects Induced by the Anticancer Drug Topotecan on the Human Topoisomerase I – DNA Complex

**DOI:** 10.1371/journal.pone.0010934

**Published:** 2010-06-03

**Authors:** Giordano Mancini, Ilda D'Annessa, Andrea Coletta, Nico Sanna, Giovanni Chillemi, Alessandro Desideri

**Affiliations:** 1 CASPUR Inter-University Consortium for the Application of Super-Computing for Universities and Research, Rome, Italy; 2 Department of Biology and Centro di Bioinformatica e Biostatistica, University of Rome Tor Vergata, Rome, Italy; Griffith University, Australia

## Abstract

**Background:**

Human topoisomerase I catalyzes the relaxation of DNA supercoils in fundamental cell processes like transcription, replication and chromosomal segregation. It is the only target of the camptothecin family of anticancer drugs. Among these, topotecan has been used to treat lung and ovarian carcinoma for several years. Camptothecins reversibly binds to the covalent intermediate DNA-enzyme, stabilizing the cleavable complex and reducing the religation rate. The stalled complex then collides with the progression of the replication fork, producing lethal double strand DNA breaks and eventually cell death.

**Methodology/Principal Findings:**

Long lasting molecular dynamics simulations of the DNA-topoisomerase I binary complex and of the DNA-topoisomerase-topotecan ternary complex have been performed and compared. The conformational space sampled by the binary complex is reduced by the presence of the drug, as observed by principal component and cluster analyses. This conformational restraint is mainly due to the reduced flexibility of residues 633–643 (the region connecting the linker to the core domain) that causes an overall mobility loss in the ternary complex linker domain. During the simulation, DNA/drug stacking interactions are fully maintained, and hydrogen bonds are maintained with the enzyme. Topotecan keeps the catalytic residue Lys532 far from the DNA, making it unable to participate to the religation reaction. Arg364 is observed to interact with both the B and E rings of topotecan with two stable direct hydrogen bonds. An interesting constrain exerted by the protein on the geometrical arrangement of topotecan is also observed.

**Conclusions/Significance:**

Atomistic-scale understanding of topotecan interactions with the DNA-enzyme complex is fundamental to the explaining of its poisonous effect and of the drug resistance observed in several single residue topoisomerase mutants. We observed significant alterations due to topotecan in both short-range interactions and long-range protein domain communications.

## Introduction

**Figure 1:**
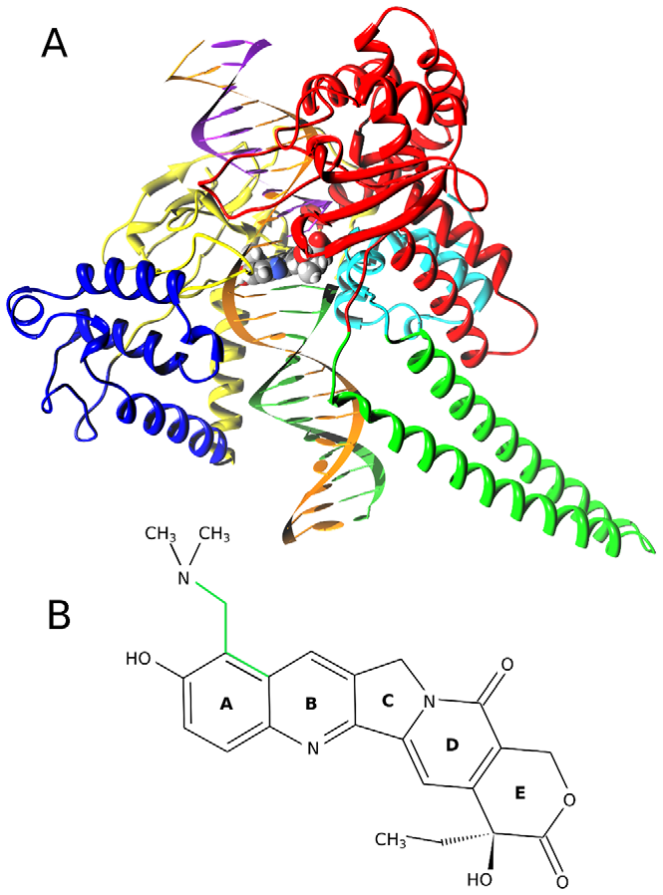
hTop1-DNA-Topotecan ternary complex structure. Panel A: Three-dimensional structure of the hTop1-DNA-TPT complex. hTop1 core subdomains I, II and III are represented in blue, yellow and red respectively, while linker and C-terminal domains in green and cyan. DNA strands are colored in orange (uncleaved strand), purple (cleaved upstream) and light green (cleaved downstream). Topotecan is represented using Van Der Waals radii and different colors for different atom types. Panel B: Topotecan chemical structure with specification of ring names. The atoms defining the dihedral angle mentioned in the “TPT and DNA motion” paragraph are highlighted in green.

Human topoisomerase I (hTop1) catalyzes the relaxation of DNA supercoils in fundamental cell processes like transcription, replication and chromosomal segregation [1], [2]. The protein is composed of 765 aminoacids residues, divided in four domains: the NH2-terminal domain (residues 1–214), the core domain (residues 215–635) which is divided in three subdomains (subdomain I: residues 215–232 and 320–433; subdomain II: residues 233–319; subdomain III: residues 434–635), the linker domain (residues 636–712) and the COOH-terminal domain (residues 713–765) (see Figure 1A) [3]–[5]. Supercoiled DNA undergoes a topological rearrangement when the catalytic Tyr723 binds the scissile strand DNA 3′ terminus thus introducing a transient break in the phosphodiester chain (catalytic mechanism B). DNA relaxation is supposed to proceed via a controlled rotation mechanism, in which the enzyme accompanies the end downstream of the cleavage site to rotate around the intact DNA strand [5].

Topoisomerase was discovered for the first time in the early 70's but extensive studies began in the 80's, when it was found that hTop1 was the only molecular target of camptothecin (CPT) [6], [7] an alkaloid with anticancer property extracted from the Asiatic plant *Camptotheca acuminata* [8]. CPT reversibly binds to the covalent intermediate DNA-enzyme, stabilizing the cleavable complex and thus reducing the rate of religation. The stalled topoisomerase I then collides with the progression of the replication fork, producing lethal double strand DNA breaks and eventually cell death [9], [10]. Due to its high toxicity, CPT cannot be used as an antitumor agent and a number of derivatives have been developed and are currently used in clinical therapy; among these, topotecan (TPT; Hycamtin, GlaxoSmithKline) is widely used to treat lung and ovarian carcinoma [11]. TPT differs from its parent compound in the oxydril and ethyldimethylammine groups on ring A (see Figure 1B) which have been observed to increase its solubility and minimize the toxicity.

Many structures of the covalent and non-covalent binary DNA-hTop1 complexes have been solved [4], [5], [12], and in all cases the NH2-terminal domain is never present, being partially unstructured and flexible. In vitro reconstituted forms of topoisomerase with only the NH2-terminal domain deleted (topo70) or both the NH2-terminal and the linker domains deleted (topo58/6.3), maintain enzymatic activity [13]. The structural dynamical properties of DNA in complex with native topoisomerase or with single mutants, displaying a varied reactivity toward CPT, have been extensively investigated by Molecular Dynamics (MD) simulations, permitting to observe a direct correlation between functional properties and inter-domain communication [14]–[19]. In particular, the computational characterization of the drug resistant A653 mutant evidenced a perturbed mobility of the linker domain that could explain the experimental higher religation activity and therefore the drug resistance [14].

In 2002 the crystal structure of the ternary complex topo70-DNA-TPT was solved [20]. TPT is found to be intercalated between the base pairs −1/+1, at the level of the cleavage site (Figure 1A), and in direct interaction with several protein residues [20]. Up to now, a single MD study on the hTop1–DNA–CPTs ternary complexes, focused on the free energy barriers for drug dissociation, has been carried out [21], but a detailed MD investigation of the structural and dynamical characteristics of the ternary complex is still lacking. Recently we have carried out a systematic investigation of the electronic properties of CPT and TPT that permitted to computationally reproduce the experimental absorption bands of the drugs [22], [23] and that ended up with the development of a reliable AMBER compatible TPT force field [24].

In the present work, taking advantage of the TPT force field, we have carried out a total of 50 nanoseconds classical MD simulation of ternary and binary complexes, interacting with the same DNA substrate present in the crystal structure of the ternary complex. Comparative analysis of the simulations indicates that topotecan produces a different behavior of the protein collective motions and an alteration in secondary structure and flexibility in regions that are not in direct contact with the drug, such as residues 633–643, between the linker and the core domains. The explanation of the dynamical role of the different chemical groups involved in the interaction with TPT has been also provided.

## Methods

### Initial configurations and simulation protocol

The initial configuration of hTop1, in covalent complex with a 22 base pair linear double helix DNA substrate, has been modeled from the crystallographic structures of the binary and ternary complexes (PDB 1K4S and 1K4T, respectively) [20]. For the binary complex the starting positions for residues 201–631 and 708–765 have been obtained from the 1K4S crystal structure and those for residues 632–707 from the 1K4T crystal structure (since the linker domain is not resolved in the former), following a mass-weighted fit of backbone atoms on 1K4S (RMSD between the two structures was 0.7 Å after the fit). The ternary complex was modeled using atomic positions from the 1K4T structure and assuming the lactone form of topotecan [23], [24]. The 22 base pair DNA sequence of the ternary complex was used in both systems (nucleotides in relevant positions in the binary system were mutated using the rotamers module present in the Chimera package). The systems have been modeled using the AMBER03 all-atom force field [25] implemented by Sorin and Pande [26] in the GROMACS MD package version 3.3.3 [27]. The protein has been immersed in a rectangular box (93×110×130 Å^3^) filled with water molecules described by means of the TIP3P rigid potential [28]. Na^+^ counter-ions have been added to neutralize DNA-enzyme complex total charge using the genion tool of the GROMACS package, which randomly substitutes water molecules with ions at the most favorable electrostatic potential positions. The resulting systems, were composed of 9456 protein atoms, 1400 DNA atoms, 40271 water molecules, 20 Na^+^ ions and one TPT molecule in the ternary complex, for a total of 131727 and 131669 atoms in the ternary and binary systems respectively. Electrostatic interactions have been taken into account by means of the Particle Mesh Ewald method (PME) [29], [30] using a cutoff of atom for the real space and Van der Waals interactions. The LINCS algorithm [31] was used to constrain bond lengths and angles. Relaxation of solvent molecules and Na^+^ ions was initially performed keeping solute atoms restrained to their initial positions with decreasing force constants of 1000 and 500 kJ/(mol • nm), for 3000 ps. The two systems have then been simulated for 25 ns with a time step of 2.0 fs and the neighbor list was updated every 10 steps. Temperature was kept constant at 300 K using the Berendsen's method [32] with a coupling constant of 0.1 ps during sampling, while pressure was kept constant at 1 bar using the Parrinello-Rahman barostat [33] with a coupling constant of 1.0 ps during sampling.

### Analysis of trajectories

Root mean square deviations (RMSD) were calculated using the following formula (after a mass-weighted least square fitting to a reference structure):
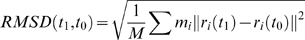
where *M* is the sum of atomic masses, *m_i_* is the mass of atom *i* and *t = 0* refers to the selected reference structure. The per-residue root mean square fluctuations (RMSF) were computed using the following equation:
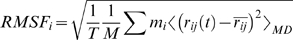
where the averages have been calculated over the equilibrated MD trajectories.

Principal components analysis (PCA) [34], [35] was carried out on the 3N×3N cartesian displacement matrix whose elements are calculated as:
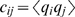
where N is the number of Cα protein or C5′ DNA atoms of the two systems and *q_i_* is the (mass-weighted) displacement of the *i-th* Cα protein or C5′ DNA atoms from the reference value (after removal of rotational and translational degrees of freedom). The first few eigenvectors of the diagonalized covariance matrix usually account for a major fraction of the total variance and projection of atomic trajectories over the corresponding eigenvectors represents large collective atomic motions. To compare the two simulations, sampled structures were clustered using the GROMOS method: after the construction of the MxM RMSD matrix (were M is the number of structures sampled). The structure with the largest number of neighbors (i.e. configurations within the cutoff range) is taken as the centroid of the first cluster and it is eliminated by the pool with all its neighbors; the process is repeated until all structures have been assigned to a cluster.

The cosine content (*c_i_*) of a principal component *p_i_* is a good indicator for good simulation sampling [36]. It ranges between 0 and 1 and is calculated in the following way:
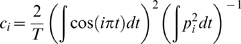
where t and T are instantaneous and total simulation time, respectively. High values of *c_i_* (close to 1) are indicative of random diffusion motion and therefore insufficient sampling [36].

All analyses have been carried out with standard tools present in the GROMACS MD package v. 3.3.3 or in-house written codes, except for secondary structure assignment, which was performed by means of the DSSP program [37]. Graphs have been obtained with the Grace program [38] and images have been created using the VMD [39] and Chimera [40] packages.

## Results and Discussion

### RMSD and secondary structure

Structural modifications of the hTop1-DNA complex during the 25 ns long simulations, in presence or absence of topotecan, can be monitored by measuring the deviation of each structure from the starting crystallographic coordinates after a superposition on the protein Cα atoms. The root mean square deviations (RMSD) value reaches an average value of about 3 Å in both simulations during the first nanoseconds, showing strong oscillations during the whole simulation time (see Figure S1 in Supporting Documents; black and red dotted lines refer to the binary and ternary complex simulations, respectively). Upon elimination of the linker domain from the analysis, the RMSD of the core and C-terminal domains has average values of 1.7 and 1.8 Å (see black and red full lines in Figure S1) and stable plateaus are reached in both systems well before 3 nanoseconds of simulation time. All results presented in the following sections refer to the last 22 nanoseconds of simulation (unless otherwise specified).

**Figure 2:**
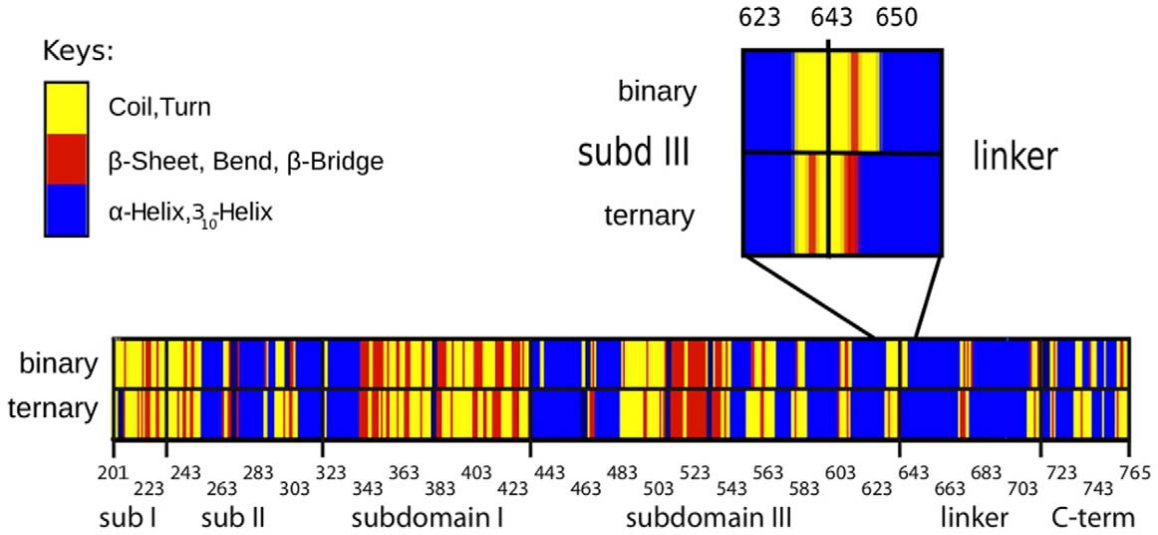
Secondary structure assignment for the binary (top rectangle) and ternary (bottom rectangle) complex. An inset is present to highlight the partial destructuration of residues 635–645 in the binary linker domain.

The average protein secondary structure was calculated over 22000 frames uniformly extracted over the 22 ns of sampling of both simulations. The results, shown in Figure 2, indicate that small structural alterations are observed only in regions far from the drug binding site. The regions around residues 283, 303 and 705, in fact, show a loss of α-helix structure when the drug is present. On the other hand, residues 633–645, at the N-terminal region of the linker domain, maintain a helical structure in the ternary complex that is lost in the binary system (see inset in Figure 2).

### Protein-DNA and protein-TPT interactions

**Figure 3:**
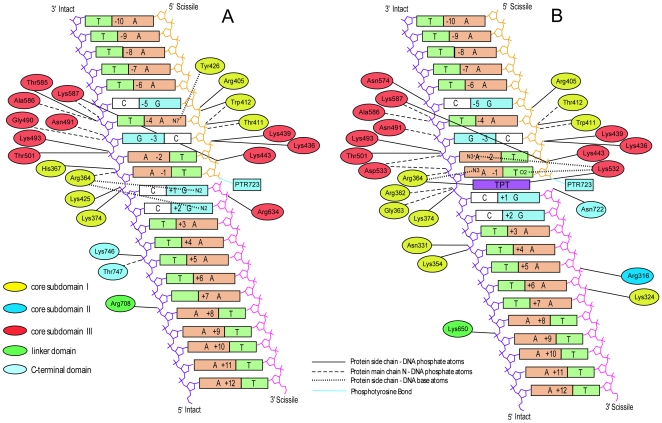
Schematic representation of the protein-DNA hydrogen bonds present for more than 75% of the simulation time for the binary and ternary complex (panel A and B, respectively). Full line, protein side chain- DNA phosphate group; dashed line, protein main chain–DNA phosphate group; dotted line, protein side chain-DNA base atoms; dash-dotted line. The phosphotyrosine bond is represented by a full cyan line. Different colors refer to different protein domains. TPT is represented in purple color.

A global picture of the protein-DNA interactions is shown in Figure 3, where the direct hydrogen bonds of the binary and ternary complexes (panel A and B, respectively) are shown when present for more than 75% of simulation time. Most of the hydrogen bonds between hTop1 and DNA found in the crystallographic structures of the binary and ternary complex (PDB 1K4S and 1K4T, respectively) [20] are well conserved during the 22 nanoseconds of MD sampling. In the X-ray structure of the ternary complex TPT is stabilized in the binding site by large DNA base stacking interactions (380 Å^2^) and by hydrogen bonds of the phosphodiester group, between the −1 and +1 base pairs in the DNA intact strand, with the main chain nitrogen atoms of Arg362 and Gly363 and the lateral chain of Lys374 [20]. As a consequence, the distance between the 5′SH (present in the modified −1 DNA base) and the phosphorous of the 3′ phosphotyrosine is 11.5 Å in the ternary complex, while only 3.5 Å in the binary one. In the simulation, the TPT-DNA stacking interactions are fully maintained, as well as the Arg362, Gly363 and Lys374 hydrogen bonds with the DNA intact strand, that are present for the 100% of the simulation time. The average distance between 5′OH (which replaces the crystallographic SH group) and the 3′ phosphotyrosine is 8.6 and 4.5 Å in the ternary and binary complex simulations, respectively. The availability of the 5′OH group also permits the direct interaction with the lateral chain of Asp722 (98% of the simulation time), that in the crystallographic structure interacts with TPT via water mediated contact.

The interactions between Arg364 with the N3 atom of the adenosine in position −1 on the uncleaved strand and of Lys532 with the O2 of −1 thymine on the cleaved strand, observed in 1K4T, are conserved in the ternary complex simulation (for more than 95% of the sampling). Thr718 is bridged via a water molecule to the phosphodiester group of the cleaved strand guanosine in position −1 for the 71% of the simulation time, while in the crystallographic structure the same residues are bridged by a direct hydrogen bond.

**Figure 4:**
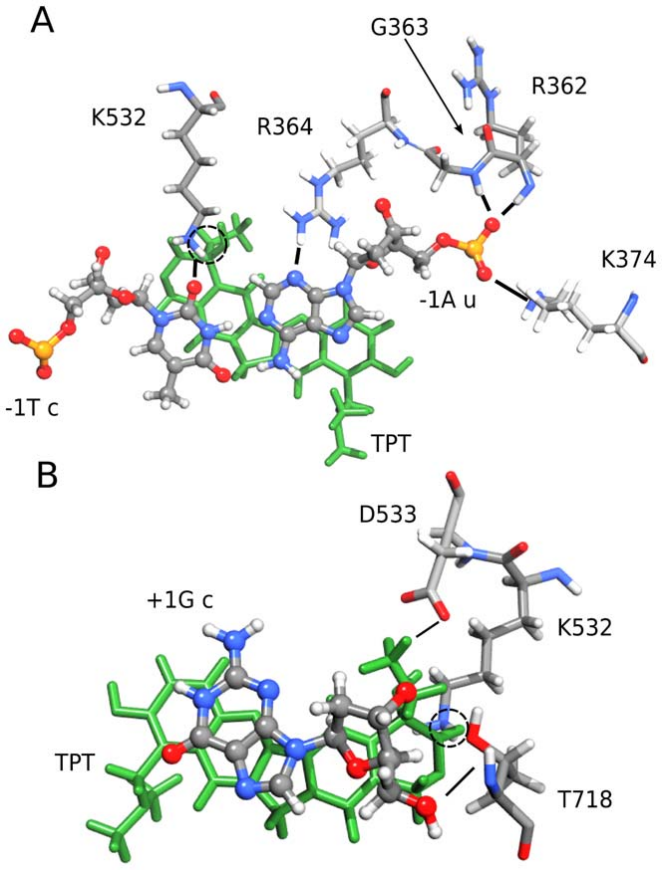
Snapshots of the drug binding pocket. Hydrogen bonds between hTop1, DNA and Topotecan. Aminoacids and nucleotides are colored according to atom types while the drug is shown in green; ball-and-stick style has been used for DNA and licorice for Topotecan and protein residues and black lines or circles (for interactions between K532 and TPT) indicate hydrogen bonds cited in the text. Panel A: View of the active site looking downstream of the cleaved strand. Panel B: View of the active site looking upstream of the cleaved strand.

In the crystal structure a single residue, Asp533, is observed to have a direct interaction with the oxydril oxygen on topotecan ring E. In the simulation such an interaction is substituted by a water mediated contact, present for 84% of simulation time (see Figure 4 for a representative snapshot of the topotecan binding pocket in the simulation). Two other residues, Lys532 and Arg364, known for their key role in the catalytic cycle [41], are in direct contact with the topotecan drug during the simulation. The side chain nitrogen of Lys532 forms a direct hydrogen bond (99% of sampling) with either the carbonylic or the oxydril oxygen atom of topotecan ring E, maintaining the distance between the Lys532-Nζ and the +1G-O5′ atoms at 8.8 Å in the ternary complex, while it oscillates between 2/3 different conformations (from 2.8 Å to 9.7 Å) in the binary one (Figure S2 in Supporting Documents). This residue is believed to act as a basic catalyst in the religation reaction, accepting a proton from the +1 cleaved 5′OH group, and permitting the oxygen nucleophilic attack on the scissile phosphate to (re-)join the two ends of the DNA backbone [42]–[44]. Therefore, in the ternary complex this critical residue remains far from its DNA target and is unable to participate in the religation reaction, while in the binary complex it visits distances compatible with a proton transfer reaction. Note that the 1K4T and 1K4S crystallographic structures have a thio-2′ deoxy-base phosphonic acid in position +1 and therefore the distance between the atoms Lys532-Nζ and +1OH5′ cannot be compared with the one in our simulation.

The side chain of Arg364 forms two direct hydrogen bonds with the B and E rings of topotecan, both present for 100% of simulation time. This result can explain the experimental finding that mutation of the nearby Gly363 to cysteine produces an enzyme resistant to camptothecin, likely causing a different orientation of Arg364 lateral chain with the consequent destabilization of the interaction with topotecan [45].

### Collective Motions

**Figure 5:**
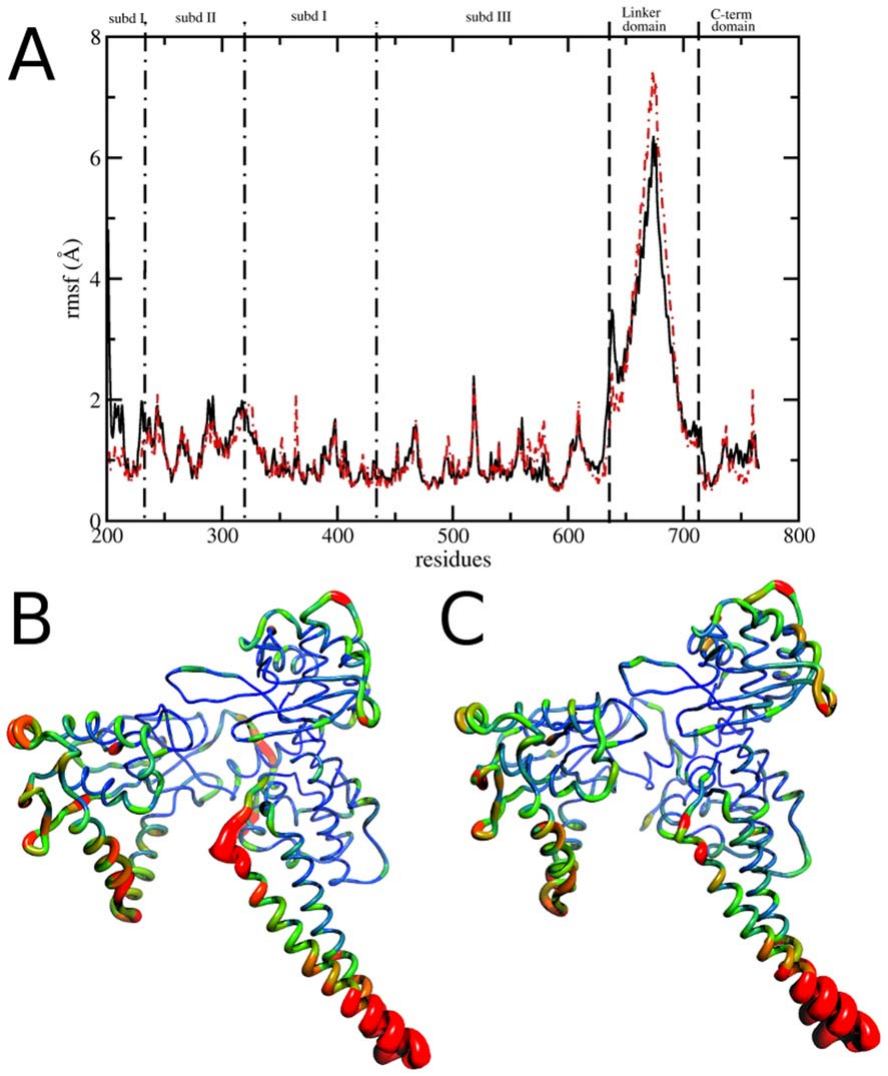
Collective motions: per residue RMSF represented with 2D and 3D figures. Panel A: Per-residue RMSF of binary (full black line) and ternary (red dot-dashed line) trajectory. Panels B and C: Representation of per-residue B factors calculated from the binary complex (Panel B) and ternary complex trajectory (Panel C). Ribbon width and color scale (from blue to red) is proportional to B factor.

**Figure 6:**
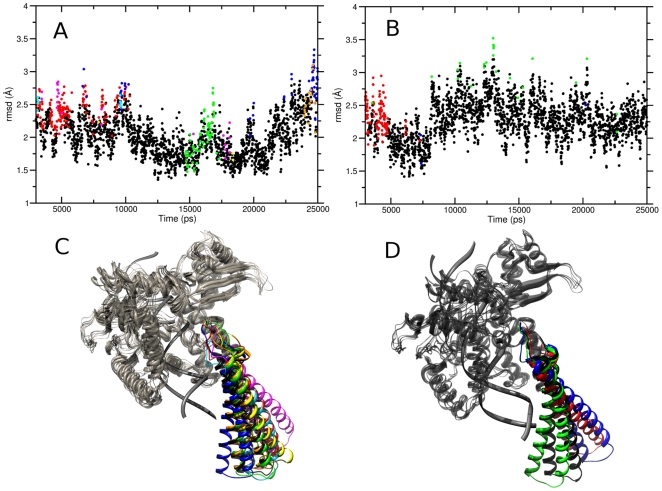
RMSD from the starting structure of the 2000 linker domain conformations used in the cluster analysis in the binary and ternary complex trajectories (Panel A and B, respectively). Visualization of the cluster centroids of the two trajectories (binary and ternary complex in Panel C and D, respectively). Each cluster is indicated with a different color.

A comparison of the per-residue root mean square fluctuations (RMSF) calculated from the binary and ternary complex trajectories shows that the two systems undergo very similar fluctuations (see Figure 5 panel A). The linker domain is the most flexible region in both systems, as expected, but residues 655–680, in the central portion of the domain, are more flexible in the ternary system (RMSF maximum values are 6.3 Å and 7.3 Å for binary and ternary complex, respectively). These differences can be well appreciated comparing panel B and C in Figure 5, where the colour and width of the ribbons are directly proportional to the RMSF. On the other hand, a larger flexibility is observed in the binary system for residues 633–643, the region connecting the linker to the core domain (RMSF max of 3.4 Å compared to 2.4 Å when topotecan is present). The same observation can be made for Arg364 that, besides the interaction with the DNA observed in both systems, forms two very stable hydrogen bonds with topotecan in the ternary complex. The higher mobility of residues 633–643 in the binary complex suggests the presence of an increased number of orientations of the linker domain, hypothesis confirmed by a cluster analysis of the configurations visited by this domain in both simulations (Figure 6). A total of 2000 structures of the 88 linker Cα atoms have been clustered after being extracted every 10 ps (5000 steps) to ensure absence of correlation between the frames. The analysis shows that the conformational space visited by the linker in the native system is strongly reduced by the presence of topotecan, since fewer clusters are visited by the ternary system and the conformations in these have a higher degree of similarity (i.e. a lower inter-cluster RMSD). In detail, the number of clusters is different in the two systems, being 8 and 4 in the binary and ternary systems, respectively, when using 1.6 nm as a cut-off value of the intercluster RMSD. Moreover, the pairwise RMSD in the binary complex is evenly distributed around 1.75 nm with a width of 1.2 nm while in the ternary complex the distribution has a peak at 1.0 nm and a width of 0.75 nm. The linker domain, therefore, visits several potential energy minima in the binary complex with comparable stability during the simulation, while its motion is more confined in the ternary complex trajectory. The evolution of clusters visited by the two simulations as a function of time is reported in Figure 6, where the RMSD from the starting structure is plotted using a different color for each family. The different flexibility of the linker domain in the two systems is apparent also by the superimposition of the 3D structures of each cluster centroid, in Figure 6, C and D.

**Figure 7:**
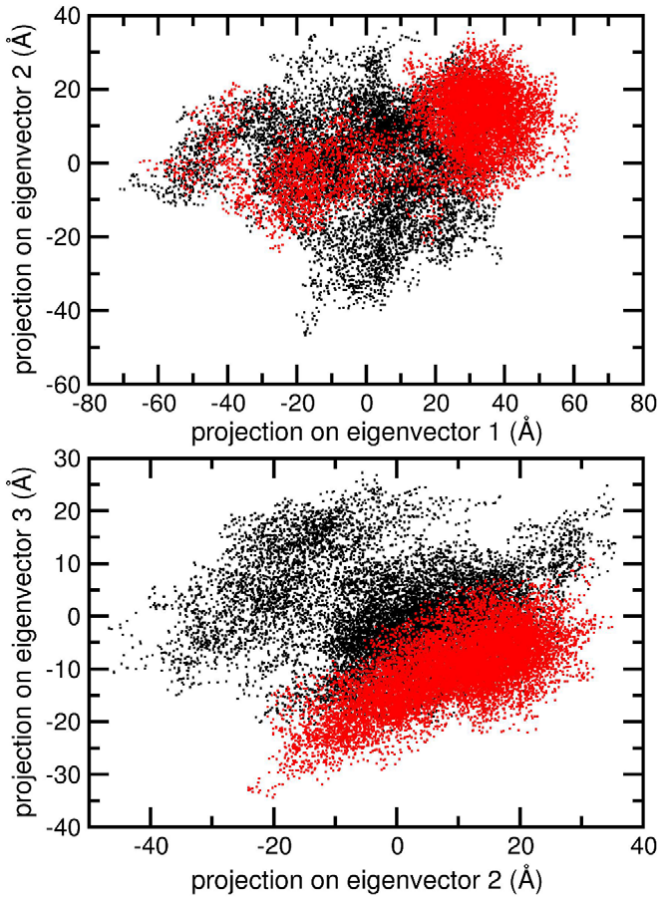
Projection of the binary and ternary complex trajectories on the subspaces formed by binary complex simulation eigenvectors. Binary and ternary complex frames are represented as black and red dots, respectively. Panel A: Projection on first and second eigenvector. Panel B: Projection on second and third eigenvector.

A picture of the global protein correlated motions has been obtained by a principal component analysis (PCA) carried out on the 565 Cα atoms of the protein. The overall fluctuations in both simulations are well described by the first three eigenvectors, which account for 60% and 70% of the total variance in the binary and ternary system, respectively. Cosine contents along these eigenvectors have maximum values of 0.05 and 0.06 for the binary and ternary complex, respectively, indicating a satisfactory convergence of simulations along these principal components. The projection of the Cα trajectories on the plane defined by the first and second eigenvectors (Figure 7A) indicates that the binary complex has a wider conformational basin than the ternary complex. In fact the displacement along the second eigenvector is more confined for the ternary complex, being 85 Å and 50 Å in the binary and ternary complex, respectively. An even stronger confinement of the ternary complex trajectory is observable in the projection on the plane formed by the second and third eigenvectors (Figure 7B). The binary complex shows two quite spread basins of comparable density, while the ternary complex is confined in a single conformational basin.

**Figure 8:**
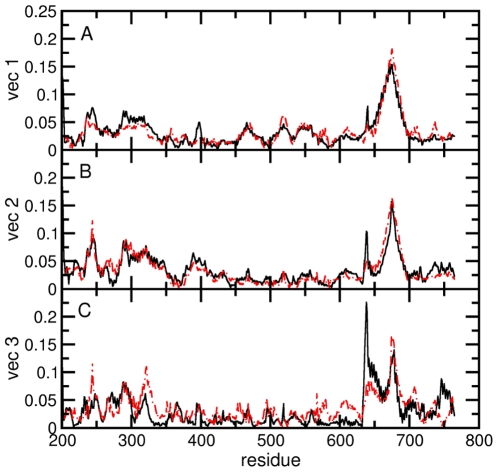
Individual weights of each Cα atom in the first three principal components (Panels A–C) of the binary (black continuous line) and ternary (red dash-dotted line) complex simulations, respectively.

In order to better understand the cause of the reduced conformational space visited by the protein in the ternary complex, the contribution of each residue to the first three eigenvectors has been calculated for both systems (Figure 8). The linker domain (and mainly residues 655–680) dominates the correlated motions in the first two eigenvectors in both systems (Figure 8, A and B). Residues 633–643, the region connecting the core with the linker domain, give a remarkable contribution to the first two eigenvectors in the binary complex, larger than in the ternary complex. Moreover, the contribution of this region to the third eigenvector is the predominant one in the binary but not in the ternary complex (Figure 8, C).

Taken together these results indicate that the presence of topotecan gives rise to an overall reduction of the conformational space of the enzyme-DNA complex, mainly due to the reduction of the subdomain III-linker junction flexibility.

Other regions are perturbed by the presence of the drug. The loop connecting the linker with the C-terminal domain and helix 21 in the C-terminal domain (corresponding to residues 703–713 and 732–741, respectively) show an increased correlated motion along the first eigenvector. The contrary is observed in the 237–250 (core subdomain II), the 287–338 and the 394–400 (core subdomain I) regions. The second region contains the so-called nose cone helices: helix 5 in core subdomain II and helix 6 in core subdomain I. It is interesting to note, therefore, that the nose-cone helices, known to play a role in the control of the DNA relaxation step [5], have stronger correlated motions along the first eigenvector in the binary complex as compared with the ternary one.

### TPT and DNA motion

As already said, TPT in the ternary complex shows strong stable interactions with DNA, (hydrophobic stacking interactions with both the −1 and +1 bases) and with the protein (several direct and water mediated hydrogen bonds). An interesting feature of the TPT molecule concerns the mobility of the di-methyl-amino group on the A ring, that is altered in the enzyme-DNA binding pocket, as compared to the simulation of TPT in water solution [24]. This can be observed by calculating the probability distribution of one dihedral angle formed by di-methyl-amino atoms (highlighted in green in Figure 1B). In the ternary complex simulation two peaks, centered at −98 and −74 degrees, are observed (Figure S3 in Supporting Documents) with a predominance of the first one, while in water solution the di-methyl-amino group visits only the second one (compare black and red lines in Figure S3). In line, the value of the dihedral angle in the two TPT crystals of the ternary complex displays values close to −98 degrees (blue dashed line in Figure S3).

**Figure 9:**
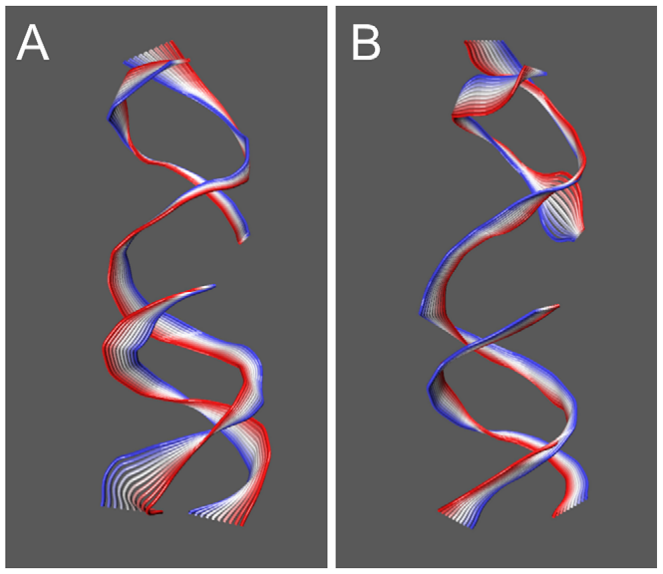
Representation of ten projections, in different colors, of the MD motions along the first eigenvector obtained from PCA analysis for the binary and ternary complex simulations (Panel A and B, respectively).

TPT also alters the DNA dynamics. In particular, an increased fluctuation of the upstream region in both the cleaved and uncleaved DNA strands is observed when compared to the binary system, while the opposite is found for the downstream region of both strands. Since in the downstream DNA region the scissile DNA strand rotates around the intact one during the relaxation process, the high rigidity observed in the presence of the drug is in line with the blocking effect imposed by the drug itself. This behaviour can be appreciated by looking at the projection of the motion along the first eigenvector obtained from PCA analysis on the C5′ DNA atoms (compare Figure 9 panel A and B for the binary and ternary systems, respectively).

### Conclusions

Detailed understanding of TPT/DNA-hTop1 interactions is an important step towards the comprehension of the causes underlying the TPT poisonous effect. An important contribution in this direction has been provided by the X-ray diffraction study of the TPT/DNA-hTop1 ternary complex [20]. In this work we have carried out MD simulations in order to investigate the dynamical properties of the TPT/DNA-hTop1 ternary complex. One of the most interesting results obtained by comparing the binary and ternary complexes trajectories is the conformational space reduction caused by the interaction with the drug, as evidenced by both the cluster (Figure 6) and PCA analyses (Figures 7 and 8). The reduced linker domain flexibility in the ternary complex is caused by the altered motion of residues 633–643 (connecting the linker to the core domain) that are less mobile than in the binary complex (Figure 5A). It is interesting to note that the internal fluctuations of residues composing the linker domain are actually slightly larger in the ternary than in the binary complex (Figure 5B–C). However the conformational distribution is mainly influenced by the 633–643 region that determines the orientation of the linker itself. This result explains the absence of the 633–707 region in the X-ray diffraction of the binary complex, that is on the other hand observed in the ternary one [20]. The linker domain flexibility has been already described to be a key element in determining the CPT resistance of Ala653Pro mutant [14]. The presence of the drug has also a direct influence on DNA flexibility since the cleavage downstream region is more rigid in the ternary complex than in the binary one, providing another possible explanation for the inhibitory effect of the drug on the relaxation process. TPT is firmly bound to DNA during all the simulation by means of stacking interactions with −1/+1 DNA base pairs and by direct and water mediated hydrogen bonds with the protein. The distance between the catalytic residue Lys532 Nζ atom and the O5′ oxygen of the G+1 base, sampled during the simulation, oscillates between 2.8 and 9.7 Å in the binary simulation while in the ternary the two groups are always separated by more than 8 Å, making it unable to act as a basic catalyst in the religation reaction. Note that this distance cannot be observed in the crystallographic experiments because the 5′OH group of the nucleotide in position +1 on the cleaved strand is substituted with a SH- group. Other direct interactions between the enzyme and TPT are formed between Arg364 and the B and E rings of topotecan, thus explaining the CPT resistance obtained after mutation of the nearby Gly363 to cysteine [45]. On the other hand also the protein-DNA complex exerts some influence on the TPT dynamics, since the rotation of the substituent on TPT ring A is altered in the ternary complex, as compared to simulations of the drug in water.

## Supporting Information

Figure S1 Root mean square deviation (RMSD) from the starting structure plotted as a function of simulation time. RMSD of the binary and ternary complexes are shown in black and red dotted lines, respectively. RMSD calculated without the linker domain contribution are represented in black and red full lines for the binary and ternary, respectively.
(0.31 MB TIF)

Figure S2 Distance between the catalytic residue Lys532 Nζ atom and the O5′ oxygen of guanidine in position +1 on the cleaved strand, represented as a function of time. Black dots: binary complex simulation; red dots: ternary complex simulation.
(0.31 MB TIF)

Figure S3 Probability distribution of dihedral angle (defined in Figure 1B) values calculated over the MD simulation of TPT in explicit water solution (red line) and in the ternary complex (black line). Vertical blue dashed lines indicate experimental X-ray values obtained from 1K4T structure.
(0.11 MB TIF)
