# Supplementary figures and images for: Structural and Dynamical Effects Induced by the Anticancer Drug Topotecan on the Human Topoisomerase I – DNA Complex
Source: PLoS One. 2010 Jun 3;5(6):e10934. doi: 10.1371/journal.pone.0010934 (PMC2880615; doi:10.1371/journal.pone.0010934)

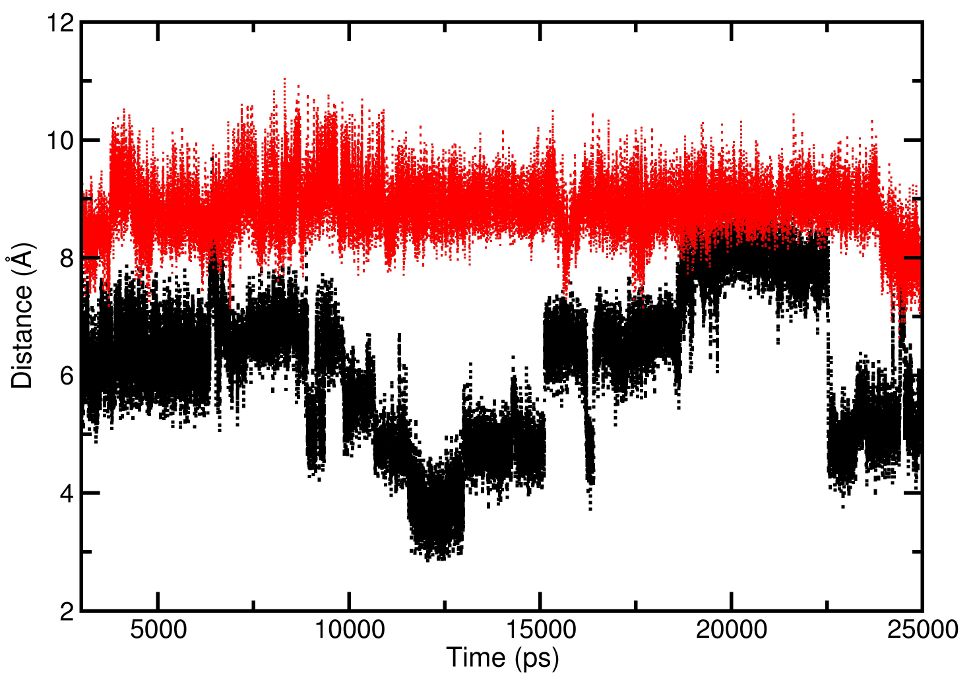

Supplement: Figure S1 — Root mean square deviation (RMSD) from the starting structure plotted as a function of simulation time. RMSD of the binary and ternary complexes are shown in black and red dotted lines, respectively. RMSD calculated without the linker domain contribution are represented in black and red full lines for the binary and ternary, respectively. (0.31 MB TIF) [file pone.0010934.s001.tif]

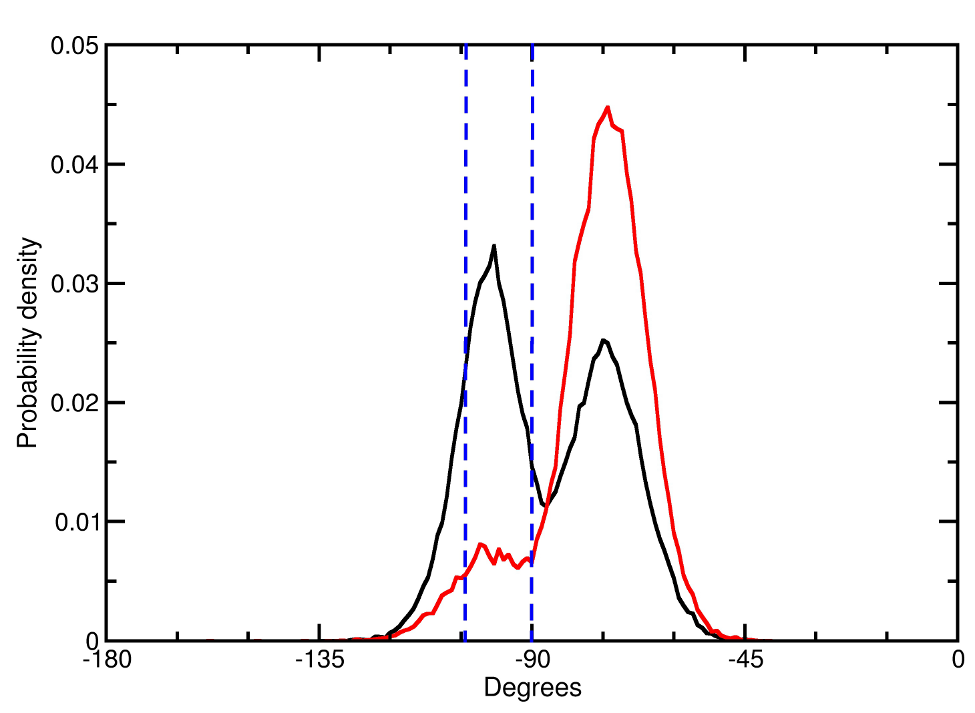

Supplement: Figure S3 — Probability distribution of dihedral angle (defined in Figure 1B) values calculated over the MD simulation of TPT in explicit water solution (red line) and in the ternary complex (black line). Vertical blue dashed lines indicate experimental X-ray values obtained from 1K4T structure. (0.11 MB TIF) [file pone.0010934.s003.tif]
